# Annual trends of human brucellosis in pastoralist communities of south-western Uganda: a retrospective ten-year study

**DOI:** 10.1186/s40249-015-0072-y

**Published:** 2015-08-31

**Authors:** Catherine Kansiime, Elizeus Rutebemberwa, Benon B. Asiimwe, Fredrick Makumbi, Joel Bazira, Anthony Mugisha

**Affiliations:** Department of Health Policy, Planning and Management, School of Public Health, College of Health Sciences, Makerere University, P.O. Box 7072, Kampala, Uganda; Department of Medical Microbiology, College of Health Sciences, Makerere University, P.O. Box 7072, Kampala, Uganda; Department of Epidemiology and Biostatistics, School of Public Health, College of Health Sciences, Makerere University, P.O. Box 7072, Kampala, Uganda; Department of Microbiology, Mbarara University of Science and Technology, P.O. Box 1410, Mbarara, Uganda; Animal Resources and Biosecurity, College of Veterinary Medicine, Makerere University School of Veterinary Medicine, P.O. Box 7062, Kampala, Uganda

**Keywords:** Trends, Brucellosis, Pastoralist communities

## Abstract

**Background:**

Human brucellosis is prevalent in both rural and urban Uganda, yet most cases of the disease in humans go unnoticed and untreated because of inaccurate diagnosis, which is often due to the disease not manifesting in any symptoms. This study was undertaken to describe trends in laboratory-confirmed human brucellosis cases at three health facilities in pastoralist communities in South-western, Uganda.

**Methods:**

Data were collected retrospectively to describe trends of brucellosis over a 10-year period (2003–2012), and supplemented with a prospective study, which was conducted from January to December 2013. Two public health facilities and a private clinic that have diagnostic laboratories were selected for these studies. Annual prevalence was calculated and linearly plotted to observe trends of the disease at the health facilities. A modified Poisson regression model was used to estimate the risk ratio (RR) and 95 % confidence intervals (CIs) to determine the association between brucellosis and independent variables using the robust error variance.

**Results:**

A total of 9,177 persons with suspected brucellosis were identified in the retrospective study, of which 1,318 (14.4 %) were confirmed cases. Brucellosis cases peaked during the months of April and June, as observed in nearly all of the years of the study, while the most noticeable annual increase (11–23 %) was observed from 2010 to 2012. In the prospective study, there were 610 suspected patients at two public health facilities. Of these, 194 (31.8 %) were positive for brucellosis. Respondents aged 45–60 years (RR = 0.50; CI: 0.29–0.84) and those that tested positive for typhoid (RR = 0.68; CI: 0.52–0.89) were less likely to have brucellosis.

**Conclusions:**

With the noticeable increase in prevalence from 2010 to 2012, diagnosis of both brucellosis and typhoid is important for early detection, and for raising public awareness on methods for preventing brucellosis in this setting.

## Background

Brucellosis is considered to be the most common zoonotic infection worldwide [1], with more than 500,000 cases recorded annually [2, 3]. In Africa, especially south of the Sahara, many of the known zoonotic diseases – including brucellosis – commonly occur and are poorly controlled in both humans and domesticated animals. The most recent review of the disease indicates that the highest incidence of brucellosis in Africa was recorded in Algeria at 84.3 per million of population per year, and the lowest in Uganda at 0.9 per million of population per year. The disease is known to be endemic in Cameroon and Ethiopia, but no specific data is available for these countries [1].

In Sub-Saharan Africa, most human cases of brucellosis go unnoticed and untreated because of inaccurate diagnosis, which is often due to the disease not manifesting in any specific symptoms. This makes it difficult to clinically distinguish brucellosis from typhoid, rheumatic fever, joint diseases and malaria [4]. For this reason, official figures do not fully reflect the actual burden, and the World Health Organization estimates that the true incidence of the disease in developing countries may be between 10 and 25 times higher than what reported figures indicate [5]. With its diverse clinical manifestations, human brucellosis can only be proven by laboratory diagnosis.

In Uganda, human brucellosis has been reported to be prevalent in both rural and urban settings [6, 7]. A recent study revealed that 12.6 % of informally marketed milk in urban Kampala was contaminated with *Brucella abortus* at purchase; and that the annual human incidence rate was estimated to be 5.8 per 10,000 people [8]. Studies on animal brucellosis have also been done in Uganda, reporting a herd prevalence of 55.6 % and an animal prevalence of 15.8 % in the pastoral dairy system in the Mbarara District [9], while higher figures of up to 100 % at herd level and 30 % at animal level were reported in the central district of Nakasongola [10]. Brucellosis is also prevalent among Ugandan wildlife [11]. Pastoral communities in Uganda are commonly found living on the periphery, adjacent to wildlife conservation parks. Additionally, their close contact with cattle, as well as their love for consuming raw milk and fermented milk products exposes them to brucellosis. While health education and promotion outreach programmes are conducted in such communities, it is not known if they are successful. Conducting a trends analysis is important to assess the effectiveness of such programmes.

The objective of this study was to describe trends in laboratory-confirmed human brucellosis cases at two public hospitals and a private clinic in the Mbarara and Lyantonde districts, in southwestern Uganda. We envisage that the data obtained will inform public health efforts for the design of effective health education programmes and development of control strategies against brucellosis in the country.

## Methods

### Study setting

Our study was conducted at three health facilities that have the capacity to diagnose and treat brucellosis: two public laboratories (the Mbarara University teaching hospital and the Lyantonde hospital), and a private clinic (the Mbarara diagnostic clinic). The retrospective study focused on the Mbarara University teaching hospital and Mbarara diagnostic clinic, which are situated less than 1 km apart. These were purposively selected because the teaching hospital serves as the referral hospital for five districts that were formerly part of the Greater Mbarara District before it was divided into five smaller districts (Isingiro, Kiruhura, Kazo, Ibanda and Mbarara). The people in these districts still access the referral hospital in the former headquarters of Mbarara. The private clinic was chosen because it had detailed records of patients that attended the clinic during our study period and had many referred patients from Mbarara hospital.

All the districts in the study area are mainly comprised populations whose livelihoods depend on cattle as well as other animal products for food and trade. They are: farmers who are settled and solely grow crops (mostly recent migrants to the area), agro-pastoralists who rear cattle as well as practice farming, and pure pastoralists or semi-nomads who rear cattle but have permanent shelters, moving their animals in dry seasons in search of water and pasture. The Lyantonde District neighbours the Kiruhura District, a predominantly cattle keeping area, and was therefore purposively selected to capture that population. This hospital receives patients from both the Lyantonde and Kiruhura districts, as well as neighbouring areas.

### Study design

A retrospective study was conducted from January 2003 to December 2012, inclusive, in order to describe trends in the prevalence of brucellosis over a 10-year period. This was done by reviewing serological reports at the Mbarara University teaching hospital and the Mbarara diagnostic clinic. This was also supplemented by a 1-year prospective study (January to December 2013), which was conducted at the Mbarara University teaching hospital and Lyantonde hospital in order to fill the gaps of the retrospective study.

### Data collection

For both the retrospective and prospective studies, brucellosis test results and socio-demographic data of the patients were obtained from laboratory records. The selected laboratories in these studies follow a standard operating procedure recommended by the Ministry of Health, Uganda. Records were searched for all suspected cases that presented to the selected clinics during the period between January 2003 and December 2012. During the prospective study, patients took brucellosis tests (January to December 2013). Recommendation by a clinician to test for brucellosis was based on the presence of clinical signs and symptoms of fever, sweating, fatigue and other symptoms as defined by the Centers for Disease Control and Prevention [12]. Individual information on sex, age, area of residence, month of diagnosis and antibody titres were obtained. Serological testing was done at the hospitals by the plate agglutination test, which has a sensitivity and specificity of 0.771 and 0.960, respectively [13]. Uganda is considered a low prevalence setting [1], therefore a dilution titre of ≥1:80 of the diagnostic test was considered positive for the disease [14].

### Data analysis

The annual prevalence rates were calculated based on the number of cases in the three laboratories as a ratio of the annual total population at risk in each facility, expressed as a percentage. The annual prevalence of human brucellosis in both studies was calculated. Descriptive analysis was conducted for retrospective data and linearly plotted to observe the trends of the disease. We stratified the analysis by health facility for the retrospective study in order to compare trends of brucellosis at a private and a public facility, as well as to guide us in future recommendations for disease control. Prospective data were analysed with Stata (Stata Corp LP, Texas, USA) version 12 statistical software package. Chi-square tests were used for categorical variables. At bivariate analysis, categorical variables including age, sex, occupation, area of residence, health facility, symptoms of brucellosis, and whether tests for other diseases such as malaria and typhoid were conducted were assessed for the association between the independent variables and presence or absence of brucellosis.

The prospective categorical data were compared using the chi-square test and *p* ≤ 0.05 was considered statistically significant. We used a modified Poisson regression model with a robust error variance to estimate the risk ratio (RR) and 95 % confidence intervals (CIs) to determine the association between the presence or absence of brucellosis and the independent variables. The advantage of using RR is that it narrows the CIs and is preferred over the odds ratio for prospective investigations [15]. The additional advantage of estimating relative risk by using a logarithm link is that the estimates are relatively robust to omitted covariates [16], in contrast to logistic regression. We adjusted the RR for other predictors or potential confounders (age and occupation) by adding them to the model that had variables with *p* ≤0.2, as determined by the bivariate analysis.

### Ethical considerations

The study protocol was approval by the Makerere University School of Public Health Higher Degrees, Research and Ethics Committee, and the Uganda National Council of Science and Technology. The study objective was explained to the directors of the hospitals and the technical staff (laboratory technicians, clinicians and nurses), while informed written consent was obtained from each participant in his/her local language. Data collected were coded for anonymity, and confidentiality was assured at all stages of the study.

## Results

### Participant characteristics

**Fig. 1:**
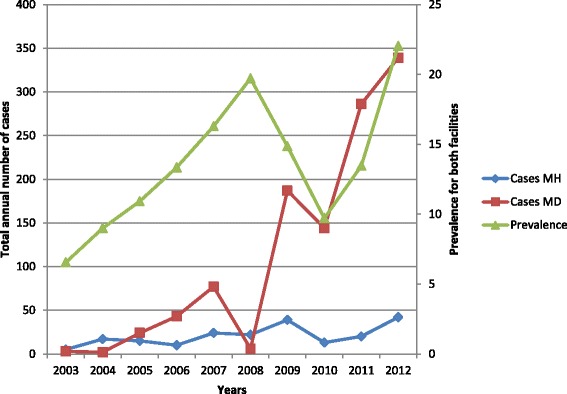
Annual trends of human brucellosis prevalence at Mbarara teaching hospital (MH) and Mbarara diagnostic clinic (MD) from 2003 to 2012

In the retrospective study, a total of 9,177 suspects (4,209 males and 4,968 females) presented to the health facilities with symptoms suggestive of brucellosis and were serologically tested. A total of 1,318 cases of brucellosis were identified using a cut-off of ≥1:80; of these, 207 (15.7 %) were diagnosed at the Mbarara University teaching hospital, while 1,111 (84.3 %) were diagnosed at the private clinic. At the teaching hospital, the number of cases per year increased from five (12.8 %) in 2003 to 17 (12.5 %) in 2004 to 24 (14.4 %) in 2007, with the highest prevalence of 42 cases (30 %) recorded in 2012. At the private clinic, the number of cases noticeably increased from two in 2004 to 77 in 2007, with a peak of 339 cases in 2012, and corresponding prevalence of 2.7 %, 17 % and 21.3 %, respectively. The most noticeable annual increase in prevalence at both facilities was observed from 2010 to 2012, with records showing a change from 13 (8.2 %) to 42 (30 %) cases at the teaching hospital (see Fig. 1), and 144 (9.9 %) to 339 (21.3 %) cases at the private clinic.

In the prospective study, a total of 610 participants presented with symptoms suggestive of brucellosis between January and December 2013, inclusive. Of these, 228 (37.4 %) were from Mbarara University teaching hospital, while 382 (62.6 %) were from Lyantonde hospital. Of the 610 participants, 194 (31.8 %) tested positive for brucellosis; 66 (34 %) were from Mbarara hospital and 128 (66 %) were from Lyantonde hospital. The annual brucellosis prevalence was calculated as the total number of cases in a given year divided by the total number of population at risk per year at the hospitals. At the Mbarara hospital, the prevalence was 66/128 (51.6 %), while at the Lyantonde hospital it was 128/382 (33.5 %).

### Gender distribution of cases

From the 1,318 brucellosis cases observed during the retrospective study, 560 were male (42.5 %) patients and 758 were female (57.5 %). To break this down further by facility, 76 (36.7 %) males and 131 (63.3 %) females were infected at the teaching hospital, while 481 (43.3 %) males and 630 (56.7 %) females were infected at the private clinic. There was no significant difference in the number of cases by gender (*p* = 0.34) at both health facilities. However, there was a significant difference (*p* < 0.001) in the number of cases diagnosed at the two health facilities, with the private clinic diagnosing more cases than the public hospital.

Of the 610 participants in the prospective study, 213 were males (34.9 %) and 397 were females (65.1 %). Of the 397 females, 144 (36.3 %) were from the Mbarara hospital and 253 (63.7 %) were from Lyantonde hospital. The infection rates among males and females at the Mbarara hospital were 22/66 (33.3 %) and 44/66 (66.7 %), while at the Lyantonde hospital they were 38/128 (29.7 %) and 90/128 (70.3 %), respectively. There were no significant differences between genders and health facilities (*p* = 0.44), or in the number of cases diagnosed at the two health facilities (*p* = 0.22), as shown by the chi-square test.

### Seasonal variation of cases

**Fig. 2:**
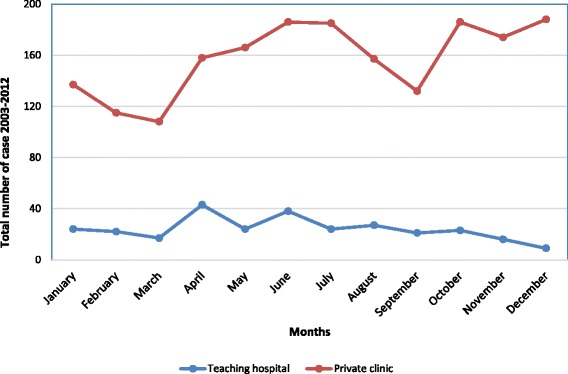
The seasonal distribution of human brucellosis at the two health facilities (Mbarara teaching hospital and Mbarara diagnostic clinic), 2003–2012

Descriptive analysis using graphs was used to show seasonal variations. The peak of brucellosis cases in the 10-year study was observed during the months of April and June at both health facilities. Cases at the private clinic increased during October, November and December, while at the teaching hospital, a marked decrease was observed in the same time period (see Fig. 2).

**Fig. 3:**
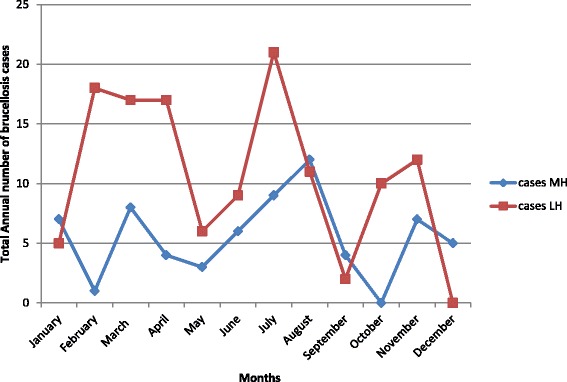
Monthly trends of brucellosis at the Mbarara teaching hospital (MH) and Lyantonde hospital (LH) in 2013

The prospective study showed that there was a significant difference in the number of cases presented at the health facilities (*p* < 0.001), with more cases observed at the Lyantonde hospital. At the Mbarara University teaching hospital, cases increased in the months of February to March, May to August, and October to November, while at the Lyantonde hospital, cases increased in the months of January to February, May to July, and September to November (see Fig. 3). A steady increase was observed at Lyantonde in May to July and at Mbarara in May to August (see Fig. 3). A decrease in the number of cases was observed in the months of April to May and November to December at both hospitals. A significant difference was observed between the health facilities and the months with the highest incidence of cases, with a *p* < 0.001.

### Symptoms of brucellosis

During the prospective study, data were collected on patients’ symptoms at presentation to the health facility. The major symptoms reported were: headaches 415/610 (68 %), joint pains 401 (65.7 %), general weakness 331 (54.3 %) and recurrent fever 323 (53 %). Other symptoms mentioned were weight loss, loss of appetite and sweats.

### Factors associated with brucellosis (prospective study)

The variables that were taken for the multivariate analysis were age, sex, occupation, health facility, symptoms of brucellosis, and whether tests were done for malaria and typhoid. The multivariate analysis showed that people with occupations such as business owner, civil servant or self-employed (RR =0.29; CI: 0.11–0.82) were less likely to have brucellosis compared to farmers and pastoralists. We adjusted the RRs for other predictors and potential confounders by adding them to the model. There were no confounding differences observed. Additionally, respondents aged 45–60 years (RR = 0.50; CI: 0.29–0.84) were less likely to have brucellosis compared to the other age groups, and those that tested positive for typhoid (RR = 0.68; CI: 0.52–0.89) were less likely to have brucellosis compared to those who tested negative (see Table 1).

**Table 1:** Multivariate analysis showing factors associated with brucellosis during the prospective study (January 2013 – February 2013)

	Brucellosis results, n (%)		Unadjusted RR(95 % CI)	Adjusted RR(95 % CI)	*P*-value
Variables	Negative 416(68.2 %)	Positive 194(31.8 %)			
Age					
0–18	38(9.1)	23(11.9)	1	1	
19–30	168(40.4)	76(39.2)	0.83(0.57–1.20)	0.77(0.53–1.12)	0.18
31–45	128(30.8)	69(35.6)	0.94(0.65–1.37)	0.86(0.58–1.26)	0.44
46–60	65(15.6)	17(8.8)	0.54(0.32–0.94)	0.50(0.29–0.84)	0.01*
+61	17(4.1)	9(4.6)	0.92(0.49–1.70)	0.83(0.45–1.51)	0.54
Sex					
Male	153(36.8)	60(30.9)	1	1	
Female	263(63.2)	134(69.1)	1.21(0.93–1.57)	0.81(0.63–1.04)	0.10
Occupation					
Farmer	142(34.1)	68(35.1)	1	1	
Agro-pastoralist	66(15.9)	41(21.1)	1.17(0.86–1.59)	0.81(0.48–1.38)	0.44
Pastoralists	14(3.4)	9(4.6)	1.19(0.69–2.05)	0.89(0.52–1.53)	0.68
Others	194(46.6)	76(39.2)	0.85(0.65–0.12)	0.66(0.39–1.12)	0.12
Malaria results					
Negative	381(91.6)	166(85.6)	1	1	
Positive	35(8.4)	28(14.4)	0.94(0.56-1.03)	2.29(0.68–1.31)	0.73
Typhoid test					
Negative	405(97.4)	120(61.9)	1	1	
Positive	11(2.6)	74(38.1)	0.67(0.53–0.87)	0.68(0.52–0.89)	<0.005*

*RR* Risk ratio

* means level of significance <0.05

## Discussion

The results of our retrospective study showed that the trend distribution of human brucellosis in the study area varied in different years, with the most noticeable increase in prevalence recorded from 2010 to 2012 at both health facilities; from 13 (8.2 %) to 42 (30 %) cases at the teaching hospital, and 144 (9.9 %) to 339 (21.3 %) cases at the private clinic. A probable explanation for this increase is that health education about brucellosis and other zoonotic diseases has been provided through local radio stations in this setting. Therefore, these awareness campaigns may be credited with helping to improve health-seeking behaviour, meaning more people present to health facilities to get tested. In contrast, a 24-year trend study conducted in Saudi Arabia found a decreasing incidence rate of disease from 26.3/100,000 in 1983–1992 to 6/100,000 in 1993–2007 [17] as a result of effective control measures in animals [18]. Therefore, in order to reduce brucellosis in humans, the disease should first be controlled in animals as they are the reservoirs of the disease [19]. This can be achieved through health education, community sensitisation and animal vaccination.

The annual prevalence of human brucellosis in 2012 was 30 and 21.3 % at the Mbarara teaching hospital and private clinic, respectively. During the 1-year prospective study, the annual prevalence was 51.6 and 33.5 % at the Mbarara teaching hospital and Lyantonde hospital, respectively. This depicts a high prevalence of the disease at these facilities. Elsewhere, in Chad [20], prevalence of human brucellosis among nomadic pastoralists was reported at 3.8 % in 2003, a much smaller prevalence. However, more often than not, it is the incidence of brucellosis that is reported, not the prevalence, which may not be an accurate representation of the situation. For instance, reported incidences are; the most recent review [1] that indicates the highest incidence of brucellosis in Africa was recorded in Algeria at 84.3 per million of population per year and the lowest was in Uganda at 0.9 per million of population per year. Therefore, there is a need to provide diagnostic tests and ensure the availability of brucellosis treatment for patients, as early diagnosis and prompt treatment is essential in preventing complications and relapses.

In both the retrospective and prospective studies, there were more females diagnosed with brucellosis than males, however, we found no significant difference between gender and having the disease at the bivariate analysis. A study in Kampala found that being female was a risk factor for brucellosis seropositivity [7]. This finding may be attributed to the specific gender roles in households in this setting. For example, females in our study area are more involved in the handling of milk and other dairy products, which are transmission routes for the disease. Similar findings have been reported in the United Kingdom and India [3, 19]. On the other hand, studies in Saudi Arabia, Greece and Tanzania have found that being male is related to risk of occupational exposure [17, 21, 22]. These disparities in gender are most likely related to the differences in practices, habits and occupations of the study populations, where for example, women handle dairy products while men handle meat and assist in the delivery of animals. There is a need to study gender relations at household level in brucellosis endemic areas. This will enable researchers to identify areas on which to focus health education efforts and risk factor assessment in order to control the disease in humans in Uganda.

Cases of brucellosis peaked during the months of April and June at both health facilities in almost every year of the retrospective study. This finding is similar to a study conducted in Saudi Arabia, which also observed the highest number of cases from April to June [17]. Furthermore, we observed an increase in cases in the months of October to December at the private clinic, but cases were remarkably reduced during the same months at the teaching hospital. The increase of cases might be due to the relationship between milk yields and rainfall as previously postulated in Uganda [6], which may influence the chance of brucellosis infection. However, in the prospective study, cases increased in the months of February to March, May to August, and October to November at Mbarara University teaching hospital, while at the Lyantonde hospital, cases increased in the months of January to February, May to July, and September to November. This could be due to the provision of diagnostic reagents by our study for the period of study which enabled diagnostic testing and case detection.

On the other hand, the significant difference between the number of cases at the health facilities (*p <* 0.001) was depicted in the reduction of cases at the public teaching hospital compared to the private clinic in the retrospective study (October to December), and the numerous declines from January to February, March to May and August to October at the Mbarara teaching hospital in the prospective study. This was because there was a lack of reagents and test kits due to budgetary constraints in the first half of the financial year (June to December), as mentioned by two health providers during the survey, which led to a considerable level of underreporting of the disease. We suggest that teaching and other government hospitals in endemic areas are preferentially equipped with diagnostic facilities, equipment, kits and reagents. Simple and rapid diagnostic tests such as the Rose Bengal plate test for screening for brucellosis and more specific confirmatory tests such as the serum agglutination test are needed in endemic areas [23, 24], especially where most health facilities do not have laboratories that offer more advanced testing for brucellosis. This is important for early detection, treatment and control of the disease.

In the prospective study, we found that respondents aged 45–60 years were less likely to have brucellosis compared to other age groups. This could be because the health facilities where the study was conducted receives patients from a wide range of communities, not necessarily pastoralists. However, a study done in Germany [23] on changing epidemiology of human brucellosis in laboratory diagnostic setting (1962–2005) found that age-specific incidence was highest for persons aged 60–69 years. Another study done in Saudi Arabia showed that the highest rate of brucellosis was in patients aged 40–49 years (100/100,000).

In addition, respondents whose occupations were business owners, civil servants or who were self-employed were less likely to have brucellosis compared to agro-pastoralists and farmers. This could be because these occupations do not require being in close proximity with animals, as compared to pastoralists. The pastoralists’ high consumption of raw milk and fresh cheese is another factor [25]. There is a need to continuously sensitise all agricultural and pastoral occupations about brucellosis and proper handling of dairy products in order to control the disease in this population.

A limitation of the retrospective study was incomplete information from the medical records. For example, age of patients was mostly recorded as ‘adult’ instead of the actual age, and there was no detailed information regarding area of residence, hence our analysis was limited. However, the missing information did not affect the trends analysis as we minimised this by conducting a prospective 1-year study. The unavailability of reagents for diagnosis did result in reduced numbers of patients presenting to the public hospital. We recommend that the government makes these services and whatever is required to treat brucellosis more available to the health facilities in order to meet the needs of patients.

## Conclusions

The 10-year study showed that trend distribution of human brucellosis cases at the two health facilities varied in different years, with a noticeable increase in prevalence from 2010 to 2012. The prospective study confirmed this. Diagnosis of both brucellosis and typhoid is important for early detection, instituting control measures and raising public awareness on prevention methods for brucellosis in the study area.
